# Growth genes are implicated in the evolutionary divergence of sympatric piscivorous and insectivorous rainbow trout (*Oncorhynchus mykiss*)

**DOI:** 10.1186/s12862-021-01795-9

**Published:** 2021-04-22

**Authors:** Jared A. Grummer, Michael C. Whitlock, Patricia M. Schulte, Eric B. Taylor

**Affiliations:** 1 Department of Zoology and Biodiversity Research Centre, University of British Columbia, 6270 University Blvd., Vancouver, BC V6T 1Z4 Canada; 2 Beaty Biodiversity Museum, University of British Columbia, 6270 University Blvd., Vancouver, BC V6T 1Z4 Canada

**Keywords:** Genome scan, Small-effect loci, $F_{\text{ST}}$, GO analysis, Rainbow trout, Ecomorph

## Abstract

**Background:**

Identifying ecologically significant phenotypic traits and the genomic mechanisms that underly them are crucial steps in understanding traits associated with population divergence. We used genome-wide data to identify genomic regions associated with key traits that distinguish two ecomorphs of rainbow trout (*Oncorhynchus mykiss*)—insectivores and piscivores—that coexist for the non-breeding portion of the year in Kootenay Lake, southeastern British Columbia. “Gerrards” are large-bodied, rapidly growing piscivores with high metabolic rates that spawn north of Kootenay Lake in the Lardeau River, in contrast to the insectivorous populations that are on average smaller in body size, with lower growth and metabolic rates, mainly forage on aquatic insects, and spawn in tributaries immediately surrounding Kootenay Lake. We used pool-seq data representing ~ 60% of the genome and 80 fish per population to assess the level of genomic divergence between ecomorphs and to identify and interrogate loci that may play functional or selective roles in their divergence.

**Results:**

Genomic divergence was high between sympatric insectivores and piscivores ($F_{\text{ST}}$ = 0.188), and in fact higher than between insectivorous populations from Kootenay Lake and the Blackwater River ($F_{\text{ST}}$ = 0.159) that are > 500 km apart. A window-based $F_{\text{ST}}$ analysis did not reveal “islands” of genomic differentiation; however, the window with highest $F_{\text{ST}}$ estimate did include a gene associated with insulin secretion. Although we explored the use of the “Local score” approach to identify genomic outlier regions, this method was ultimately not used because simulations revealed a high false discovery rate (~ 20%). Gene ontology (GO) analysis identified several growth processes as enriched in genes occurring in the ~ 200 most divergent genomic windows, indicating many loci of small effect involved in growth and growth-related metabolic processes are associated with the divergence of these ecomorphs.

**Conclusion:**

Our results reveal a high degree of genomic differentiation between piscivorous and insectivorous populations and indicate that the large body piscivorous phenotype is likely not due to one or a few loci of large effect. Rather, the piscivore phenotype may be controlled by several loci of small effect, thus highlighting the power of whole-genome resequencing in identifying genomic regions underlying population-level phenotypic divergences.

**Supplementary Information:**

The online version contains supplementary material available at 10.1186/s12862-021-01795-9.

## Background

Understanding the divergence of populations and the mechanisms responsible for it are important for the fields of speciation, macroevolution, ecology, and conservation [1]. In many cases, population-level divergences are associated with character displacement and phenotypic divergence of sympatric forms, such as in threespine sticklebacks (e.g., [2]) or Nicaraguan crater lake cichlids (e.g., [3]). Recent advancements in sequencing technologies have enabled investigations into the genomics underlying traits associated with phenotypic divergences in non-model systems. In some cases, few highly differentiated loci of large effect can be responsible for population-level phenotypic or behavioural divergence in spite of the majority of genomic differentiation being very low between populations as the result of either recent divergence and/or ongoing gene flow (e.g., *Heliconius* butterflies; [4, 5]).

Determining the genetic architecture of a phenotypic trait—whether the trait is controlled by few loci of large effect, many loci of small effect, or some combination thereof—is important for understanding how traits respond to evolutionary processes including selection and migration [6]. In fishes, there are a few examples of a small number of loci having an effect on phenotypic divergence between sympatric forms. For instance, Jacobs et al. [7] identified ~ 10 genomic outlier regions in brown trout (*Salmo trutta*) containing several genes involved in development, growth, and gene expression and regulation that differ between piscivorous (fish-eating) and insectivorous populations. In Atlantic cod (*Gadus morhua*), a ~ 17 MB (megabase) region containing two chromosomal inversions is associated with population divergence based on migration phenotype [8]. Additionally, in Pacific salmon (genus *Oncorhynchus*), one small genomic region has been shown to control a vital phenotype for the natural history of two species—adult migration timing [9].

Theory predicts that the distribution of allelic effects on quantitative traits should be nearly exponential, where few loci have large effects and explain most of the variance in traits, but many loci with smaller effects explain the remaining variation in the trait [10, 11]. This pattern was seen for “age at maturity” in Atlantic salmon, where two genes of large effect and > 100 genes of small effect underly this trait [12]. Across taxa, several phenotypes are explained by many loci of small effect. For instance, hundreds of genetic variants explain only ~ 10% of the phenotypic variation in human height [13]. And a genome-wide analysis of 50,000 SNPs (single nucleotide polymorphism) in the collared flycatcher (*Ficedula albicollis*) revealed that no SNPs were significantly associated with forehead patch size, a sexually selected trait [14]. Furthermore, Kardos et al. [14] report that thousands of individuals and near-complete genome sequencing are necessary to reliably detect large-effect loci.

Identifying the genomic regions underlying divergent ecological forms can provide insights into the genomic architecture of population divergence. Here, we address this question in two ecomorphs of rainbow trout (*Oncorhynchus mykiss*) in Kootenay Lake of southeastern British Columbia. Rainbow trout in this lake provide an example of divergence into co-existing insectivorous and piscivorous forms, which has repeatedly been observed in trout and char. For example, Arctic charr (*Salvelinus alpinus*) have repeatedly evolved sympatric forms of zooplanktivores and piscivores on multiple continents [15, 16], including Iceland’s Lake Thingvallavatn (e.g., [17]). Additionally, a large and piscivorous form of brown trout (“Ferox” trout; *Salmo trutta*) occurs throughout Ireland and Scotland [18, 19]. A variety of divergent life-history patterns can be found in populations across the native range of *O. mykiss*, including migration patterns (resident vs. anadromous), habitat type (lake vs. stream), and diet (piscivory vs. insectivory) [20]. Whereas genomic regions have been found that are associated with migration and habitat type [9, 21], little is known about the evolutionary mechanisms promoting divergence of piscivorous and insectivorous *O. mykiss* populations.

**Fig. 1:**
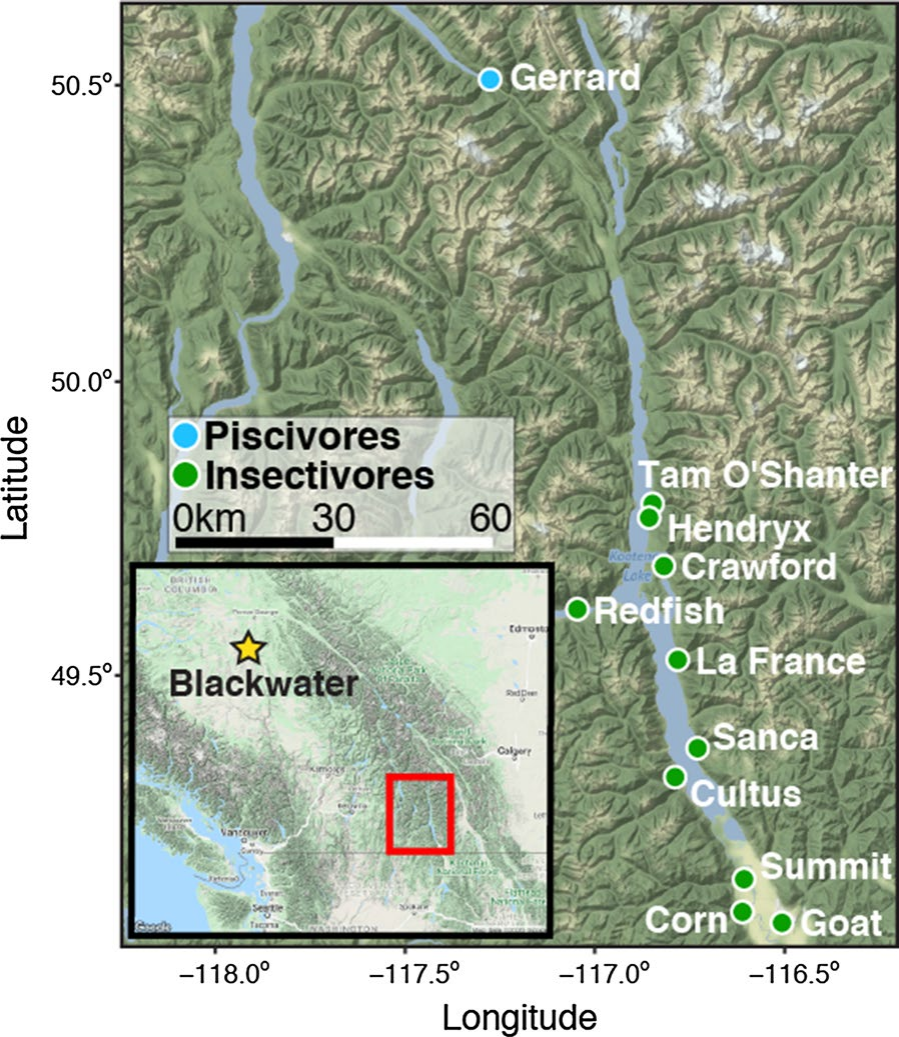
Study region of Kootenay Lake in southeastern British Columbia showing sampling locations, including the Blackwater River (shown in inset). See Additional file 1: Table S1 for more detailed sampling location information. Note that all individuals are sympatric in the main portion of Kootenay Lake during the non-breeding portion of the year

The rainbow trout ecomorphs in Kootenay Lake differ in a wide variety of ecological and life-history traits. Individuals of the large piscivorous form (often > 60 cm and 5 kg, but up to 12 kg) are locally known as “Gerrards” and have an adult dietary specialization on Kokanee salmon—small, land-locked sockeye salmon (*O. nerka*)—and spawn near the abandoned town site Gerrard (thus the name “Gerrards”) on the Lardeau River that drains into the north arm of Kootenay Lake [22, 23]. In contrast, the smaller form (typically $\le$ 50 cm and $\le$ 2 kg) is primarily insectivorous and made up of several populations that spawn in smaller tributaries that drain into Kootenay Lake [23] (Fig. 1). The piscivorous and insectivorous forms are sympatric in the lake for a majority of the year (e.g., non-breeding season) after spending their first 1–2 years separated as juveniles in their respective streams. Piscivorous individuals have significantly larger head and upper jaw sizes, likely due to their specialization on larger prey (Neufeld, BC Ministry of Forests, Lands, Natural Resource Operations and Rural Development, unpublished data). In addition to these morphological differences between ecomorphs, the piscivores have significantly faster growth rates and higher standard metabolic rates relative to those of insectivores, and are behaviourally more bold as juveniles (e.g., took less time to explore novel habitat) [24]. This pattern of differentiation is consistent with the “pace-of-life syndrome” that has often been invoked to explain the divergence of closely related populations and species [25–27]. Thus, ecology can have a direct impact on the evolution of particular life-history strategies [28].

Previous research has shown that populations of these ecomorphs in Kootenay Lake are genetically distinct at microsatellite DNA loci ($F_{\text{ST}}$ = 0.14; [76]), that no interbreeding is occurring, and that phenotypic differences between piscivorous and insectivorous forms show a high degree of heritability [20]. Together, these studies provide evidence against phenotypic plasticity, versus genetic mechanisms, as the source of this sympatric morphological polymorphism [29]. We therefore sought to answer three questions regarding the ecological and evolutionary divergence of the two *O. mykiss* forms in Kootenay Lake. First, what is the level of genomic divergence between piscivorous and insectivorous populations? Second, can we identify genomic regions responsible for the phenotypic divergence of these two forms? And third, is the phenotypic divergence between forms caused by few loci of large effect or many loci of small effect?

We addressed these questions using a whole-genome resequencing approach (“pool-seq”), which is a cost-effective technique for estimating population-level allelic frequencies and identifying loci putatively involved in functional genetic divergence of closely related populations [30]. This resulted in a dataset composed of millions of single nucleotide polymorphisms (SNPs) sampled from across the genome, with approximately 20× coverage per site. Based on the natural history of these populations, we hypothesized that elevated regions of genetic differentiation would harbour genes that fall into two classes: (1) those underlying metabolic and/or physiological processes due to body size and growth rate differences between the populations, and (2) genes associated with determining feeding behaviour and/or faciocranial morphology in relation to the divergent diet between populations (e.g., [31, 32]).

## Results

### DNA sequence quality and genomic alignments

To address our hypotheses, we performed pool-seq on three populations—piscivores from Kootenay Lake, insectivores from Kootenay Lake, and insectivores from the Blackwater River (see Fig. 1 for map). Each of the three pools (*n* = 80 individuals per population) produced ~ 290 million raw read-pairs, with the majority (83–87%) passing quality filters (Table 1). The percentage of the genome covered following quality control and coverage thresholds was 62–63% with mean depth of coverages at 23–24× (Additional file 1: Fig. S1). Sequencing coverage varied across chromosomes, with the chromosomal ends often having higher sequencing coverage (Additional file 1: Fig. S2). Levels of genome-wide nucleotide diversity ($\pi$) also showed a similar pattern with elevated levels on ends of chromosomes (Additional file 1: Fig. S3), matching well to regions of known partial tetrasomy retained from the salmonid-specific whole-genome duplication event (Ss4rR; [33, 34]). In the dataset including all three populations, 8,140,802 SNPs met all filters and were included in analyses, whereas the Kootenay Lake piscivores-insectivores dataset had 6,896,554 SNPs. The density of SNPs varied across the genome, with areas of lower density occurring in centromeric regions (Additional file 1: Fig. S4).

**Table 1:** Sequence read data for the three groups of rainbow trout in this study

	Piscivores	Insectivores	Blackwater
No. reads	592,736,824	574,066,240	590,881,476
No. reads discarded	62,411,176	51,402,968	66,731,872
No. duplicate reads removed	28,161,256	23,892,440	33,282,020
No. reads passing QC	502,164,392	498,770,832	490,867,584
No. reads mapped	365,737,358	364,938,474	363,369,176
Proportion of genome covered	0.628	0.633	0.635
Mean coverage after filters	23.16 ± 5.42	23.06 ± 5.33	24.14 ± 5.86

### Population differentiation

**Fig. 2:**
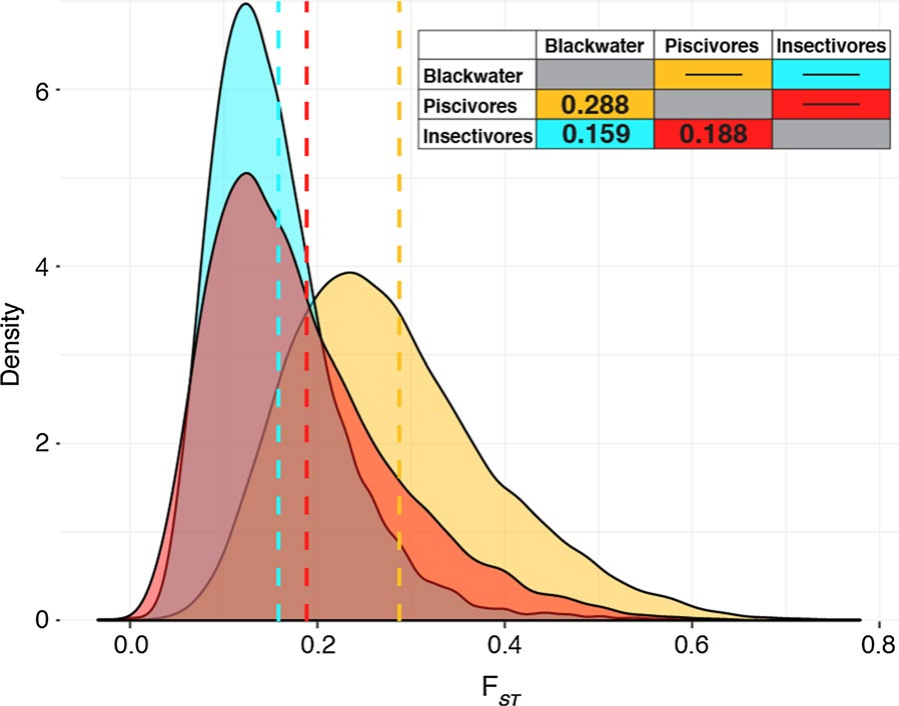
Distributions of 100 kbp non-overlapping window Weir and Cockerham $F_{\text{ST}}$ estimates, 19,512 in total, between all pairs of rainbow trout groups. Orange represents Blackwater insectivores–Kootenay Lake piscivores, blue is Blackwater insectivores–Kootenay Lake insectivores, and red is Kootenay Lake insectivores–Kootenay Lake piscivores. The table shows mean values of each comparison, which are drawn with dashed lines in the graph

We estimated the fixation index $F_{\text{ST}}$ both on 100,000 bp non-overlapping windows and single SNPs to identify genomic regions putatively under selection and estimate the overall level of population differentiation between piscivorous and insectivorous ecomorphs. Two methods to estimate $F_{\text{ST}}$ were used, and the Weir and Cockerham $F_{\text{ST}}$ estimates were highly congruent with the PoPoolation2 (“Karlsson”) results; 19 of the 20 most divergent windows shared and overall pairwise estimates very similar (results not shown). For consistency, only Weir and Cockerham $F_{\text{ST}}$ estimates are presented. Our non-overlapping window $F_{\text{ST}}$ analysis revealed high differentiation between all three populations (Fig. 2). The genome-wide divergence was higher between sympatric piscivores and insectivores ($F_{\text{ST}}$ = 0.188) than between Kootenay Lake insectivores and Blackwater River insectivores ($F_{\text{ST}}$ = 0.159), a population that is > 500 km (straight line) from Kootenay Lake. Although the modes of the Blackwater–Kootenay insectivore and Kootenay piscivore–Kootenay insectivore $F_{\text{ST}}$ distributions are approximately the same (Fig. 2), the latter distribution is right-skewed, indicating a larger fraction of the genome has higher divergence in the sympatric population-pair. The Kootenay Lake piscivores and Blackwater River trout were the most divergent populations with an overall $F_{\text{ST}}$ = 0.288.

**Fig. 3:**
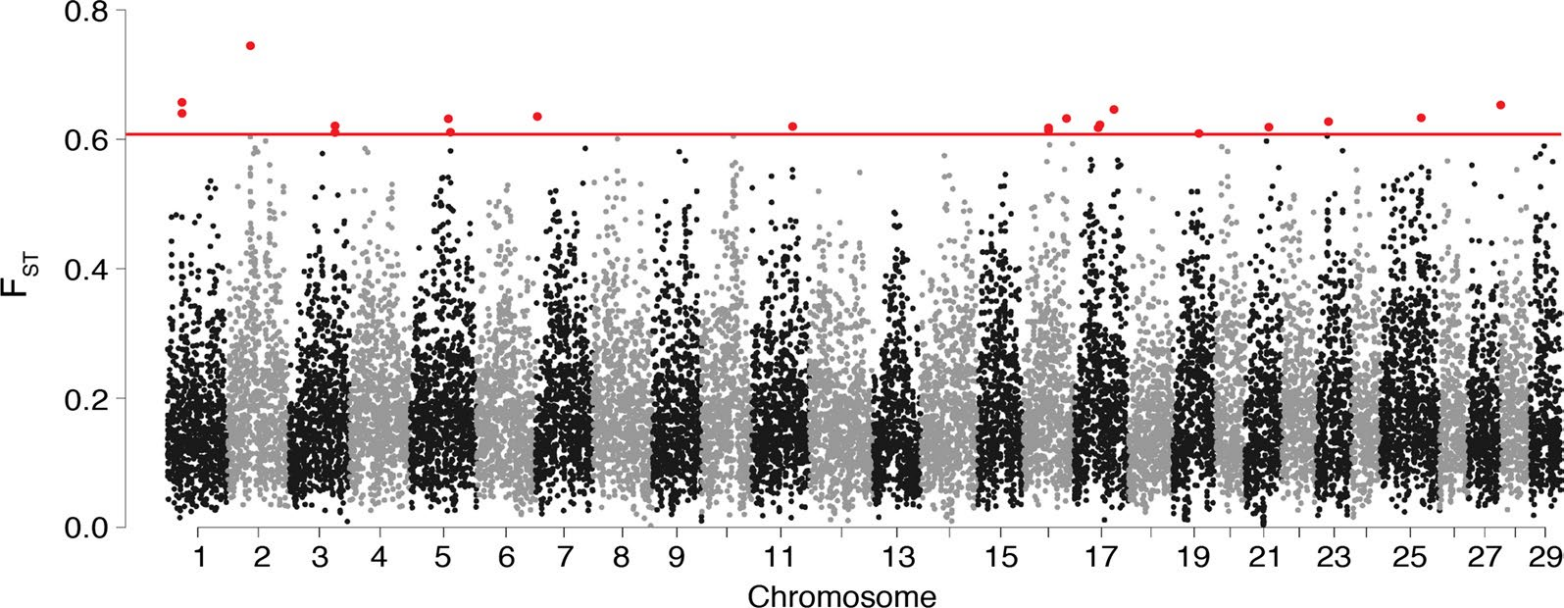
Manhattan plot of 100 kbp non-overlapping window-based $F_{\text{ST}}$ estimates between piscivorous and insectivorous rainbow trout from Kootenay Lake. The “outlier” cutoff line is shown in red, along with the windows that were above this value (0.609)

### Highly differentiated genomic regions

Two methods were used to identify highly differentiated (“outlier”) genomic regions: non-overlapping $F_{\text{ST}}$ windows and the “local score” approach described by Fariello et al. [35]. Although single-SNP-based estimates of $F_{\text{ST}}$ reached 1.0 in the empirical data (Additional file 1: Fig. S5), the maximal window-based $F_{\text{ST}}$ estimate was 0.746 on chromosome 2 (Fig. 3). In general, the high overall level of genomic differentiation between piscivores and insectivores did not lead to obvious “islands of differentiation” surrounded by regions of low differentiation. The 20 most divergent windows (representing the most divergent 0.10% of all windows) all had $F_{\text{ST}}$ estimates > 0.609, and were spread across 13 chromosomes (Additional file 1: Table S2).

To examine whether selection or recombination was driving signals of high genetic differentiation, we compared patterns of within-population nucleotide diversity ($\pi$) to absolute differentiation (*d*$_{xy}$) across four bins containing windows of increasing genetic divergence ($F_{\text{ST}}$ > 0.2, 0.3, 0.4, and 0.5). In both piscivores and insectivores, $\pi$ estimates were lower in the bins containing regions of higher genetic differentiation (Table 2). The decrease between the genome-wide and $F_{\text{ST}}$ bin > 0.5 $\pi$ estimates was ~ 73% in the piscivores, whereas the same decrease was only ~ 23% in the insectivores. No pattern existed, however, for *d*$_{xy}$ across the bins of increasing genetic divergence (Table 2).

**Table 2:** Estimates of within population genetic diversity ($\pi$) and absolute genetic differentiation between populations (*d*$_{xy}$) of piscivores and insectivores by genomic bin

Bin	$\pi _{Pisc}$	$\pi _{Ins}$	*d*$_{xy}$
*F*_ST_ > 0.5	0.000557	0.00224	0.00216
*F*_ST_ > 0.4	0.000719	0.00241	0.00205
*F*_ST_ > 0.3	0.00108	0.00255	0.00205
*F*_ST_ > 0.2	0.00151	0.00269	0.00209
Genome-wide	0.00209	0.00289	0.00218

**Table 3:** Results from GOrilla gene ontology (GO) analysis of genes in the windows with $F_{\text{ST}}$ estimates $\ge$ 0.5, arranged by *p*-value (smallest to largest)

GO term	Description	*p*-value
GO:0042574	Retinal metabolic process	9.88e$^{-05}$
GO:0048008	Platelet-derived growth factor receptor signaling pathway	1.49e$^{-04}$
GO:0050731	Positive regulation of peptidyl-tyrosine phosphorylation	2.18e$^{-04}$
GO:0030029	Actin filament-based process	3.28e$^{-04}$
GO:0007158	Neuron cell–cell adhesion	3.78e$^{-04}$
GO:0048589	Developmental growth	3.92e$^{-04}$
GO:0051279	Regulation of release of sequestered calcium ion into cytosol	4.38e$^{-04}$
GO:0060020	Bergmann glial cell differentiation	4.63e$^{-04}$
GO:0040007	Growth	5.02e$^{-04}$
GO:0070895	Negative regulation of transposon integration	5.89e$^{-04}$
GO:0070894	Regulation of transposon integration	5.89e$^{-04}$
GO:0048697	Positive regulation of collateral sprouting in absence of injury	5.89e$^{-04}$
GO:0048696	Regulation of collateral sprouting in absence of injury	5.89e$^{-04}$
GO:0035264	Multicellular organism growth	6.51e$^{-04}$
GO:0030036	Actin cytoskeleton organization	6.57e$^{-04}$
GO:0007010	Cytoskeleton organization	7.08e$^{-04}$
GO:0090148	Membrane fission	7.28e$^{-04}$
GO:0016079	Synaptic vesicle exocytosis	9.66e$^{-04}$
GO:0019730	Antimicrobial humoral response	9.66e$^{-04}$

**Fig. 4:**
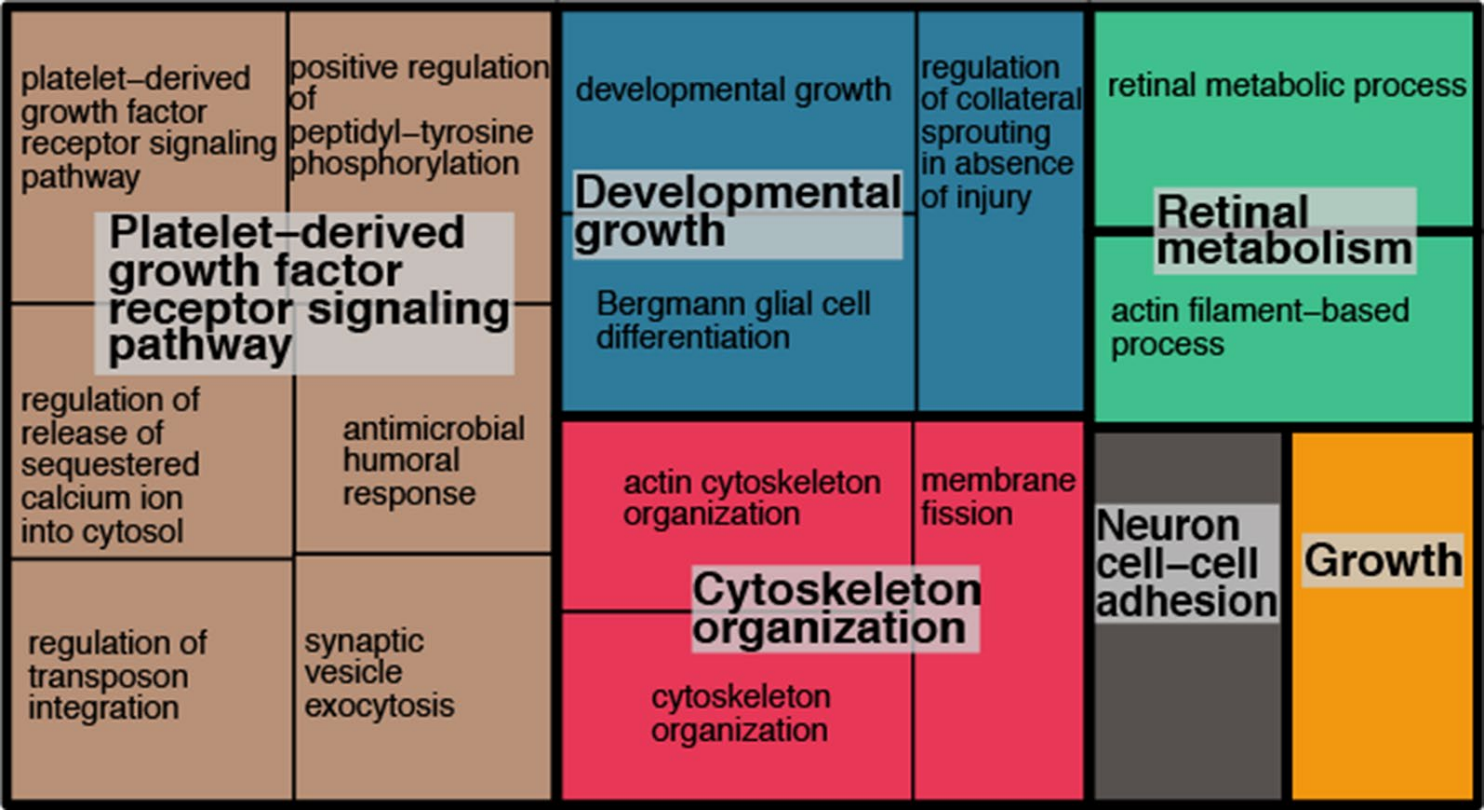
Results from a gene ontology (GO) enrichment analysis of loci from the most divergent 100 kbp windows ($F_{\text{ST}}$ > 0.5, *n* = 187) between piscivorous and insectivorous rainbow trout from Kootenay Lake visualized as a “treemap” when the target gene set is not included in the background set. Processes are grouped by functional class (colour), and box size of each process is scaled by the significance (– log$_{10}$ *p*-value; see Table 2) of enrichment of that process in relation to all processes in the genome. E.g., a larger tile size indicates a more significant result

Terms match those in Fig. 4

Given that the local score approach has not been assessed for accuracy and rates of false-positive detection (only power; see [35] for details), we performed simulations with parameters mimicking our empirical dataset, and chose the tuning parameter ($\xi$) to equal the 85% quantile of the − log$_{10}$ *p*-value distribution (further details below in "Methods"; see Additional file 1: Figs. S6–S7 for *p*-value distributions of FLK and LK test statistics on simulated data). Assuming a chromosome-wide error correction rate of 0.05 ($\alpha$) and 30 chromosomes in a genome (the rainbow trout “version 1” reference genome has 29), the local score test would be predicted to incorrectly identify significant regions (e.g., false positives) on 1–2 chromosomes per genome. In our simulations of only neutral loci, ~ 6 regions per genome on average were identified as significant outliers (5.58 for FLK and 5.50 for LK; Additional file 1: Table S3), leading to a type I error rate of 19% for the FLK test and 18.3% for the LK test. Although FLK and LK are closely related statistics, approximately 7% of the outlier regions were significant based on the results of one test (either FLK or LK), but not the other.

According to a binomial test and false-positive rates calculated from the simulated data, we did not observe more significant local score results than expected by chance in either the FLK (*p* = 0.0533) or LK tests (*p* = 0.8094) in the empirical data, meaning that these numbers of significant genomic regions could be all false-positives. Due to the high false-positive rate from this test and weak power to differentiate neutral from “outlier” regions, we did not use the local score to identify outlier regions in our empirical data. We instead focus on divergent regions identified from the $F_{\text{ST}}$ analysis.

### Gene functions in highly differentiated genomic regions

After identifying highly differentiated genomic regions, we sought to determine the biological functions of genes showing strong divergence and determine if they support or refute our hypotheses. We determined biological processes of genes through gene ontology (GO) analyses, where we partitioned our data by level of genomic divergence (genes in windows with $F_{\text{ST}}$ > 0.2, 0.3, 0.4, 0.5; and the 20 most divergent 100 kb windows). According to our hypotheses, we should see genes related to growth, metabolism, and/or faciocranial development become overrepresented in the more divergent genomic bins.

The rainbow trout genome has annotations for 42,884 protein-coding genes and 71,223 mRNAs. After filtering, our dataset had sequence data from 90% of all annotated genes (38,532) and 93% of all mRNA transcripts. The gene ontology analysis of genes (*n* = 47) in the 20 windows with the highest $F_{\text{ST}}$ estimates did not produce any statistically significant results, likely due to too few genes included in the analysis. Gene ontology analyses of the four bins containing $F_{\text{ST}}$ estimates > 0.2, 0.3, 0.4, and 0.5 resulted in 95, 72, 22, and 19 GO terms/processes, respectively (Fig. 4; Additional file 1: Figs. S8–S10). Whether the target set of genes was included in the background set made no difference on which GO terms were identified (results not shown). Although GO terms for behaviour and growth are present in the bin of least divergent loci ($F_{\text{ST}}$ > 0.2; Additional file 1: Fig. S8), they are among 90+ other biological processes. However, we observed the process of “behaviour” in the $F_{\text{ST}}$ > 0.4 bin (among 21 other processes; Additional file 1: Fig. S10), and more noticeably, growth and developmental growth in the bin of most divergent loci as two of 19 processes (Fig. 4; Table 3). Furthermore, the GO term for retinal metabolism, which includes the regulation of genes involved in development and growth, was also enriched in genes in the most divergent genomic regions. Other significantly enriched processes in the most divergent genomic regions ($F_{\text{ST}}$ > 0.5) included blood vessel formation (“platelet-derived growth factor receptor signaling pathway”), cellular-level assembly of the cytoskeleton (“cytoskeleton organization”), and neuron cell-cell adhesion.

We also searched annotations of the five most divergent $F_{\text{ST}}$ windows, and serine/threonine-protein kinase BRSK2-like was found on chromosome two in the window with the highest $F_{\text{ST}}$ estimate (Additional file 1: Table S4). This gene functions to regulate insulin secretion in fish, potentially playing a role in differential growth and development between ecomorphs. Lastly, based on gene annotations, we observed no evidence of the functional importance of genes associated with morphological feeding adaptations between these two populations.

## Discussion

Population divergence resulting from divergent selection on distinct phenotypes has been documented in many cases [36–38]. The genetic mechanisms underlying ecologically relevant traits, however, are generally not well understood. Here, through a dataset consisting of millions of genome-wide SNPs, we identified divergent loci involved with cell growth and differentiation that are associated with the divergence of two ecomorphs of rainbow trout—piscivores and insectivores. The genetic control of these ecomorphs does not seem to be controlled by a few loci of large effect, but rather by many genomic regions that are highly diverged between the populations. Our results further indicate that these two populations are moderately to highly divergent from one another with a genome-wide $F_{\text{ST}}$ estimate of 0.18, suggesting little to no gene flow between these populations. These results are in agreement with previous research with microsatellite DNA loci that has shown that the two phenotypes are quite divergent from one another ($F_{\text{ST}}$ = 0.14; [39]), and that these ecomorphs are largely genetically controlled [20].

### Differential growth implicated in divergence of sympatric ecomorphs

Rainbow trout, as well as salmon (both Atlantic and Pacific species), are economically important species in the aquaculture industry that produces meat for human consumption. The Food and Agriculture Organization of the United Nations estimated an average annual production of ~ 800,000 tonnes and > $3 billion from world aquaculture production of *O. mykiss* alone [40]. Accordingly, much research has been done to determine the genetic mechanisms determining growth and muscle mass (e.g., “fillet weight”) in these species (e.g., [41, 42]). Genetic association studies indicate that body weight in *O. mykiss* is a polygenic trait, with the most important genomic regions typically explaining < 5% of the genetic variance (e.g., [43, 44]). Two previous studies on *O. mykiss* have identified loci on chromosomes 21 [44] and 27 [43] that explain 2.5 and 1.7%, respectively, of the genetic variance in body weight. One of the 20 most divergent $$F_{\text{ST}}$$ windows we identified on chromosome 21 is less 150 kb from the region identified by Neto et al. [44].

We hypothesized that genes either involved with growth and related metabolic processes or faciocranial morphology and feeding behaviours would underly the divergence of sympatric rainbow trout ecomorphs in Kootenay Lake. Identifying single SNPs or genes in the most divergent genomic regions as causal variants related to these traits is difficult given the high genome-wide divergence we estimated between insectivorous and piscivorous populations. Nonetheless, a gene that plays a role in the regulation of insulin secretion—serine/threonine-protein kinase BRSK2-like (100% similar to 987 bases of mRNA sequence)—was found in the most divergent $F_{\text{ST}}$ window [45]. In teleost fishes, insulin is involved in somatic growth, reproduction, and development [46, 47], indicating that it could play a key role in differential growth between these two populations. This was the only gene of potentially large effect that we found in support of our hypothesis.

In addition to inspecting individual genes for their potential roles in population-level divergence, we also looked at sets of genes and their roles in emergent biological functions through gene ontology (GO) analyses. We hypothesized that biological functions and processes likely playing a role in the divergence of these ecomorphs would make up a greater proportion of the more strongly differentiated loci. In the least divergent bin of genomic regions that we analyzed ($F_{\text{ST}}$ $\ge$ 0.2), 238 GO terms were found spanning a variety of biological functions and processes (Additional file 1: Fig. S8). In contrast, only 19 significantly enriched GO terms occurred in the most divergent genomic regions ($F_{\text{ST}}$ $\ge$ 0.5), and growth functions accounted for more than half (10/19) of these GO terms. Interestingly, under the same diet, piscivores’ growth and standing metabolic rates during ontogenetic development significantly increase relative to those of insectivores [24], corroborating our genetic results here. The gene ontology results indicate that the piscivorous phenotype is controlled by many loci of smaller effect and provide support to our hypothesis that growth plays a key role in leading to ecological divergence between populations.

Beyond the “growth” and “developmental growth” GO terms, gene regions associated with “retinal metabolism” were also significantly enriched in the most divergent $F_{\text{ST}}$ bin. Retinoids (including retinal and other vitamin A derivatives) have two physiological functions—visual pigments in the eye and hormonal retinoids that regulate the expression of target genes involved in embryonic development, differentiation, and postnatal growth [48, 49]. Regulated amounts of retinoic acid and retinol are needed, particularly within mammalian embryonic tissues, for normal cell differentiation, proliferation, and morphogenesis [50]. Though less is known about the roles of these molecules in fishes, it is conceivable that they play analogous physiological roles and therefore could help explain some of the morphological differences between insectivores and piscivores.

Although not part of our search strategy, we identified via BLAST a combination of four transforming growth factor-$\beta$ family genes that were present in multiple, un-linked chromosomal regions in the top 20 $F_{\text{ST}}$ windows. We were not able to confirm, however, that these sequences indeed are from these genes (versus an unidentified repeating element in the rainbow trout genome) and therefore code for these growth proteins. Interestingly, a recent study examining the evolutionary origin of piscivory in brown trout (*Salmo trutta*) also identified transforming growth factor-$\beta$ genes associated with the evolution of piscivory [7]. It is possible that the unidentified fragments in our BLAST searches represent paralogs or isoforms of proteins in the TGF-$\beta$ superfamily. The common ancestor of all salmonids underwent a whole genome duplication event approximately 125 million years ago (“salmonid-specific fourth vertebrate whole-genome duplication, Ss4R”; [51]), in addition to a teleost-specific whole genome duplication event approximately 300–350 million years ago [52, 53]. Therefore, the current rainbow trout genome assembly may be lacking annotations of growth factor paralog isoforms that we have potentially identified in outlier regions. Genes controlling morphological traits are not well annotated as well, potentially explaining why we did not find any such genes significantly diverged between populations. Furthermore, this could be because our approach for detecting outliers is not optimal for detecting loci of small effect.

### Evolutionary divergence of sympatric *O. mykiss* forms

Populations of piscivores and insectivores have been sympatric in Kootenay Lake for at most ~ 15,000 years, since the end of the last glacial maximum. It is not known whether these populations diverged in sympatry or allopatry and subsequently established sympatry, but it is assumed that they evolved in Kootenay Lake and did not originate there via human introduction (some human-mediated introductions of Gerrard trout have occurred to other areas for angling purposes). The high divergence we estimated between these populations ($F_{\text{ST}}$ = 0.18) implies a long period of isolation, potentially pre-dating postglacial colonization. However, population divergence estimates between trout populations are often high, even for geographically proximate populations (e.g., strong IBD or IBE [isolation-by-environment]; [54]). Conversely, high genome-wide $F_{\text{ST}}$ estimates can result from population bottlenecks/founder effects that cause reductions in genetic diversity in either or both populations. Although Kootenay Lake is large, the piscivorous population is small with an estimated census size of ~ 300–1000 spawning individuals recorded annually since 1961 [55]. Indeed, we observed on average lower levels of nucleotide diversity in piscivores than in insectivores across most of the genome (Additional file 1: Fig. S3). However, the higher levels of nucleotide diversity we observed in insectivores may be because this ecomorph was sampled from multiple populations around Kootenay Lake, which likely increased estimates of $\pi$.

With a high overall genome-wide divergence and strong background differentiation between ecomorphs, such as we see in this system, finding clear candidate loci of large effect or “genomic islands” harbouring genes potentially involved in causing population divergence is difficult. In some cases, a suite of genes involved in local adaptation or divergence of phenotypes may be found linked together in a non-recombining block, for instance in an inverted chromosomal region (e.g., [8, 56]). However, we did not find any such candidate genomic regions. While trying to identify genomic outliers, using only $F_{\text{ST}}$ estimates could be misleading given correlations between divergence and other features such as genetic diversity [57] and recombination [58]. Without a recombination map for wild rainbow trout, we are unable to directly account for this correlation in our analyses. We did, however, examine correlations between within-population genetic diversity ($\pi$) and absolute genetic differentiation between populations (*d*$_{xy}$), which help isolate the effects of selection from recombination. The patterns between $\pi$ and *d*$_{xy}$ (Table 2) provided further evidence for evolutionary recent selection driving the genomic divergence of piscivores from insectivores and act as further evidence against recombination being the main factor driving the overall pattern of genomic divergence between these ecomorphs.

Growth was one of the functions enriched in the most divergent loci ($F_{\text{ST}}$> 0.5) in our gene ontology (GO) analyses. This is consistent with the hypothesis stemming from the pace-of-life syndrome that closely related populations or species should differ in physiological traits that have co-evolved with the particular life histories of each population/species. This differentiation in growth-related genes could potentially underly the observation that piscivores have higher growth and standard metabolic rates than insectivores Monnet et al. [24]. Results from both our study and Monnet et al. highlight the importance of growth traits in differentiating these ecomorphs, thus producing a hypothesis regarding the functions of these genes that can be tested in an integrated physiological-genetic study.

## Conclusions

Through the analysis of pool-seq data, we demonstrated that two feeding ecomorphs of rainbow trout that are sympatric for much of their life spans in Kootenay Lake are highly genetically differentiated from each other. The ecological and evolutionary distinctiveness of these ecomorphs—including contrasting diets, non-overlapping breeding sites, distinct morphologies, physiologies, and divergent genomes—highlights the critical role of managing them separately to promote their persistence into the future. Our results demonstrate an association between genes controlling growth and highly differentiated genomic regions, indicating a potential functional role of these loci in the divergence of these two rainbow trout feeding ecomorphs. Our results inform future studies that should perform transcriptomic analyses of these growth genes to evaluate gene expression during piscivorous *O. mykiss* development and the ontogenetic shift that accompanies a changing diet from primarily invertebrates to fish [23]. Our study further indicates the potential role of growth genes in causing phenotypic and ecological divergence of natural populations.

## Methods

### Sampling and DNA extraction

Fish were sampled from three separate populations: 80 piscivores from the Lardeau River (spawning site ~ 50 km north of Kootenay Lake, BC), eight insectivores each from 10 tributaries immediately surrounding Kootenay Lake (all 80 individuals combined together as a single “insectivore” population), and 80 insectivorous rainbow trout from the Blackwater River of central British Columbia (Fig. 1; Additional file 1: Table S1). The $F_{\text{ST}}$ values estimated from microsatellite data between insectivorous populations range from 0.04 to 0.08, and all differ from the piscivorous population by $F_{\text{ST}}$ = 0.11–0.28 [39]. The Blackwater River population was used as an outgroup for identifying significantly differentiated genomic regions (see below). Spawning adult piscivores were collected by angling in the spring, with some piscivore juveniles collected in the late summer/fall in the Lardeau River. Kootenay Lake insectivores were collected via electrofishing in the late summer/early fall. Fish were collected during the spawning season to facilitate population identification. Whole genomic DNA was extracted from fin tissues, or in a few cases, dried fish scales, with a Qiagen DNeasy extraction kit (Qiagen, Valencia, CA) and quantified with a Qubit fluorometer (Life Technologies, Carlsbad, CA). DNA from 80 individuals per population was combined into a single pool (for a total of three pools) in equimolar ratios for preparation of pool-seq libraries [30], then libraries were sequenced with 125 bp paired-end reads on an Illumina HiSeq 2500 at the Génome Québec Innovation Centre of McGill University.

### Bioinformatic dataset assemblies

Raw sequence data (fastq format) were processed using the PoolParty pipeline [59] as follows. We first used FastQC [60] to assess raw read sequence quality. Raw paired-end reads were trimmed using the *trim-fastq.pl* script (part of PoPoolation v1; [61]) based on a Phred quality cutoff score of 20 and trimmed sequence length > 50 bp. We then used BWA [62] to align reads to the using the “version 1.0” *O. mykiss* reference genome (NCBI link), then Samblaster [63] and SamTools [64] were used to remove PCR duplicates and ambiguously aligned reads (e.g., reads with a low mapping quality), respectively. Reads were then sorted by coordinate and removed if unpaired in Picard Tools (v2.4.1; http://broadinstitute.github.io/picard/) and Samtools. Lastly, indel regions were then identified using the PoPoolation2 [65] *identify-genomic-indel-regions.pl* script, and SNPs were discarded if within 5 bp of the indel regions using the *filter-sync-by-gtf.pl* script in PoPoolation2. SNPs were retained that had (1) two alleles, (2) a minor allele frequency (MAF) cutoff > 0.05 globally (e.g., across all populations), (3) at least two copies of the minor allele at a locus (to mitigate the effect of sequencing errors), and (4) a sequencing depth of 15 $\le$ *x* $\le$ 50 within each population (data filtering script available in the Dryad repository). The minimum bound of 15× increases our confidence in SNP calls, while the upper bound of 50× was established to eliminate paralogs while retaining true homologs.

### Population differentiation

We used the fixation index ($F_{\text{ST}}$) to estimate population differentiation with a window-based approach. We calculated $F_{\text{ST}}$ using two different approaches: the method of Weir and Cockerham [66] (R script available in the Dryad repository), and the “–karlsson” option within PoPoolation2. Both $F_{\text{ST}}$ methods were used on two datasets, one containing only Kootenay Lake piscivores and insectivores for identifying outlier regions, and a second dataset containing the Kootenay Lake samples along with Blackwater River samples, to estimate pairwise genome-wide differentiation between the three populations. $F_{\text{ST}}$ was calculated for 100,000 bp windows (along the reference genome) with a step-size of 100,000 bp (e.g., non-overlapping windows), giving ~ 19,500 $F_{\text{ST}}$ estimates across the genome. Results by genomic position were visualized as a Manhattan plot in the R package *qqman* [67].

### Identifying genomic regions of high differentiation

Two approaches were used to identify genomic regions that were highly differentiated between piscivores and insectivores and therefore potentially responsible in underlying their phenotypic divergence. The first method was with a window-based $F_{\text{ST}}$ approach as described above (Weir and Cockerham [66] calculation, 100,000 bp non-overlapping windows). This is a reliable indicator of locus-specific estimates of population divergence, however, determining genomic outliers based solely on $F_{\text{ST}}$ estimates can result in false-positives that may arise in neutral loci due to other processes such as recombination rate variation [58]. We focused on windows with the highest 0.1% of $F_{\text{ST}}$ estimates (*n* = 20) as a first step in identifying the genomic regions that may be associated with the phenotypic differentiation of these two ecomorphs. Non-overlapping windows were used because a sliding-window approach leads to non-independent tests from overlapping windows with no clear statistical adjustment for multiple comparisons.

Outlier detection via $F_{\text{ST}}$-based approaches may lead to false-positives when identifying putative regions involved in local adaptation because $F_{\text{ST}}$ estimates can reflect patterns of recombination rather than selection [58]. In lieu of a recombination map, patterns of nucleotide diversity within populations ($\pi$) and absolute differentiation between two populations (*d*$_{xy}$, which is independent of levels of genetic diversity within each population) can provide further evidence for or against selection in highly differentiated genomic regions. For instance, when a beneficial mutation arises in a population, $\pi$ will decrease in this region as the mutation sweeps through the population. If the beneficial mutation has recently evolved, *d*$_{xy}$ will not be elevated in selected regions. However, *d*$_{xy}$ will be elevated in regions of low $\pi$ if the beneficial mutation is “ancient” (e.g., more than 10$^{5}$ generations, given a mutation rate of ~ 10$^{-8}$). We therefore compared patterns of $\pi$ and *d*$_{xy}$ in bins of genomic windows ranked by genetic differentiation, with windows divided into four $F_{\text{ST}}$ bins ($F_{\text{ST}}$ > 0.2, 0.3, 0.4, and 0.5; same as GO analyses described below), to help distinguish between the evolutionary processes (e.g., recombination or selection) affecting highly differentiated genomic regions between insectivores and piscivores. The median $\pi$ value was calculated within each population in 100 kb windows in NPStat [68, 69], and the median *d*$_{xy}$ was calculated in a custom R script included in the Dryad data repository.

As a complementary approach to outlier identification via $F_{\text{ST}}$ windows, we also used the “local score” approach developed by Fariello et al. [35]. The local score method identifies genomic regions of high differentiation between populations by using a Lindley process to identify stretches of adjacent/linked SNPs that show significant differentiation as determined by single-locus analyses. This method has the benefit over a fixed window approach in that the outlier region bounds are not user-specified and are instead determined during analysis. As in the $F_{\text{ST}}$ window-based analysis, this approach takes advantage of linkage by using divergence information from adjacent sites and is therefore superior to single-locus outlier detection methods. The local score method implements a chromosome-wide error rate correction, meaning $\alpha$ fraction (e.g., 0.05) of the chromosomes in the genome will produce false positives. We used the significance of LK (Lewontin–Krakauer) and FLK (an extension of the LK test using a population kinship matrix “*F*”; [70]) single-locus test results expressed as *p*-values that were calculated in R scripts provided by Bonhomme et al. [70], as the input for the local score analysis. We considered loci as outliers at the 0.05 significance level.

The value of the “tuning parameter” ($\xi$) is important in local score analyses, which determines the threshold for identifying regions as outliers. The local score approach assumes a uniform distribution of *p*-values (from either the FLK or LK test statistic). However, in the case of a non-uniform distribution of *p*-values, $\xi$ must be adjusted accordingly. Fariello et al. [35] state that $\xi$ must be between the mean and maximum values of the − log$_{10}$ *p*-value distribution, and we explored various values in this range. $\xi$ values close to the max(− log$_{10}$ *p*-value distribution) resulted in no outliers identified, and $\xi$ values close to mean(− log$_{10}$ *p*-value distribution) produced hundreds of significant outlier regions (results not shown). We therefore set $\xi$ in all analyses to equal the 85% quantile of the − log$_{10}$ *p*-value distribution.

One potential downside to the local score approach is that the true type I error rate (e.g., false-positive rate) has not been previously reported. We therefore estimated it through simulations mimicking our empirical dataset as follows. A single ancestral population of 1000 individuals evolved for 1000 generations and then split into three populations, each consisting of 1000 individuals. These three populations evolved independently (e.g., no migration) for 2500 more generations (generating an $F_{\text{ST}}$ estimate of ~ 0.18 between all three populations) at which point an average of 23 chromosomes (from a truncated distribution of 15–50 and $\sigma$ = 5) were sampled from each population, based on the average sequencing depth per population from the empirical data (see "Results"). Each chromosome was 75 MB in length and simulated in SLiM v3 [71, 72] with a recombination rate of 1.5 × 10$^{-8}$ (chromosome was ~ 1 Morgan in length) and mutation rate of 2 × 10$^{-7}$ to generate ~ 15 million SNPs per genome after implementing a MAF filter (> 0.05); only neutral mutations were introduced. Thirty chromosomes were then combined into a “pseudo-genome” replicate to mimic the empirical dataset (29 chromosomes), from which allelic frequencies were calculated that were then used in FLK and LK analyses using the R scripts provided in Bonhomme et al. [70]. Local score tests were then performed on the FLK and LK results of each genomic replicate using the R scripts provided by Fariello et al. [35] and results were examined at the 0.05 significance level, per genome. One hundred pseudo-genome replicates were simulated and analyzed (e.g., 3000 chromosomal replicates). To determine if significantly more genomic regions were identified in our empirical data than expected given the false discovery rate estimated in our simulations (see "Results" below), we ran an exact binomial test in R with significance determined at the *p* $\le$ 0.05 level. The null hypothesis is that we expect as many false positives in the empirical data as are found in the simulations.

### Functions of divergent loci

We performed gene ontology (GO) enrichment analyses to identify putative functions of genes in the most divergent genomic regions between piscivores and insectivores. The GO database is designed to be species-neutral, and therefore annotations were transferred from *Homo sapiens* to *O. mykiss* [73]. We looked at gene function in two sets of divergent loci. The first set contained the genes in the 20 most divergent $F_{\text{ST}}$ windows (*n* genes = 45). And secondly, by separating our data into four bins based on estimates from our window-based $F_{\text{ST}}$ analyses: windows with $F_{\text{ST}}$ estimates > 0.2, 0.3, 0.4, and 0.5. These bins represent the 37% (*n* windows = 7219), 14% (2,737), 4.4% (854), and 0.9% (187) most divergent regions in the genome, respectively. We organized our data into bins based on divergence estimates because we expected the loci associated with biological processes most likely involved in the divergence of these populations to emerge in the more highly differentiated genomic regions (e.g., the genomic bins with higher $F_{\text{ST}}$ estimates) as compared to the less differentiated genomic regions (genomic bins with lower $F_{\text{ST}}$ estimates). According to our hypothesis, the most divergent genomic regions would contain genes enriched for processes related to growth and metabolism.

We used bedtools [74] to extract gene and protein annotations from the available rainbow trout reference genome. We used the GOrilla online platform [75, 76] to identify significantly enriched biological processes in differentiated genomic regions with two slightly different methods: (1) including the target gene set (all loci in an $F_{\text{ST}}$ bin) in the background set (all genes in the rainbow trout genome), vs. (2) excluding the target gene set from the background set. GOrilla uses a minimum hypergeometric (mHG) score to assign the significance of a term occurring in the target set vs. background set of genes. The mHG score reflects the surprise of seeing a particular GO term in the target set compared to its probability of occurrence in the background set, under the null assumption that all GO terms in the background set occur with equal probability. An exact *p*-value of this score corrected for multiple testing is then calculated. All GO analyses were performed with a significance threshold of *p* $\le$ 0.001 (as recommended by the authors). Gene ontology analysis results were visualized with Revigo [77] as “treemaps”, where the size of each functional category was scaled by its – log$_{10}$ *p*-value (e.g., a smaller *p*-value equals a larger tile size). Beyond the GO analyses, we also used annotations of genes in the five most divergent $F_{\text{ST}}$ windows to identify plausible candidates underlying the phenotypic divergence of insectivorous and piscivorous forms.

## Supplementary Information

**Additional file 1.** Additional tables and figures.

## Data Availability

Raw, un-edited DNA Sequences are available in NCBI’s Short Read Archive, BioProject ID PRJNA695019. Sampling locality information is available in Additional file 1: Table S1. Scripts for analysis are available in the Dryad repository (Dryad link; https://datadryad.org/stash/share/jYk11SUp6azAZKMNbbdwt3JRZLe-fVdtmLcTby0vbTQ).
