# Supplementary material for: Growth genes are implicated in the evolutionary divergence of sympatric piscivorous and insectivorous rainbow trout (Oncorhynchus mykiss)
Source: BMC Ecol Evol. 2021 Apr 22;21:63. doi: 10.1186/s12862-021-01795-9 (PMC8063319; doi:10.1186/s12862-021-01795-9)
Supplement: Supplementary file 1 — Additional file 1. Additional tables and figures. [file 12862_2021_1795_MOESM1_ESM.docx]

Additional figures and tables for “Growth genes are implicated in the evolutionary divergence of sympatric piscivorous and insectivorous rainbow trout (*Oncorhynchus mykiss*)”, by Grummer *et al.*.


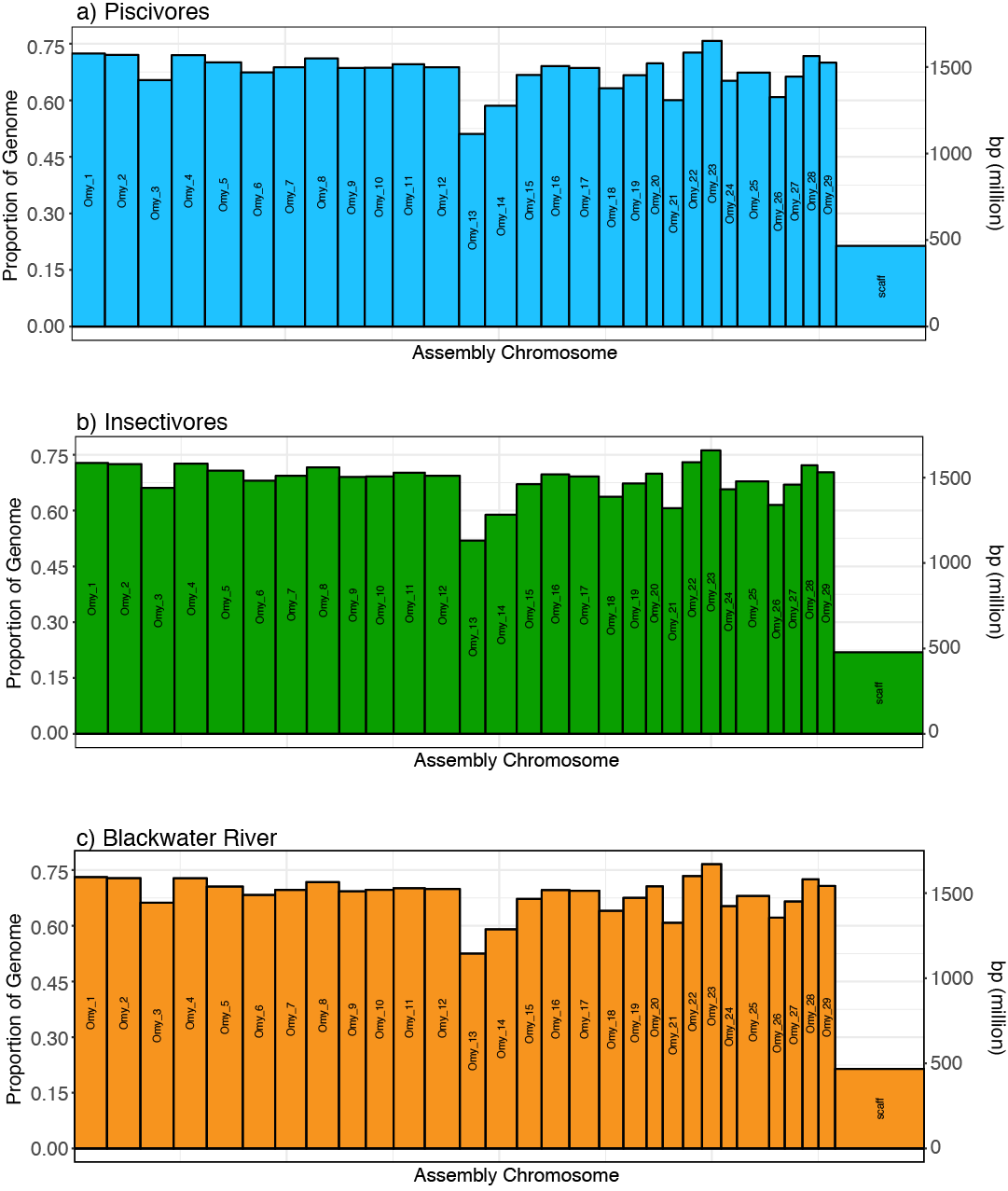


Figure S1. Sequencing coverage by chromosome following data filtering and quality controls for Kootenay Lake (panels (a) and (b)) and Blackwater River (c) rainbow trout.


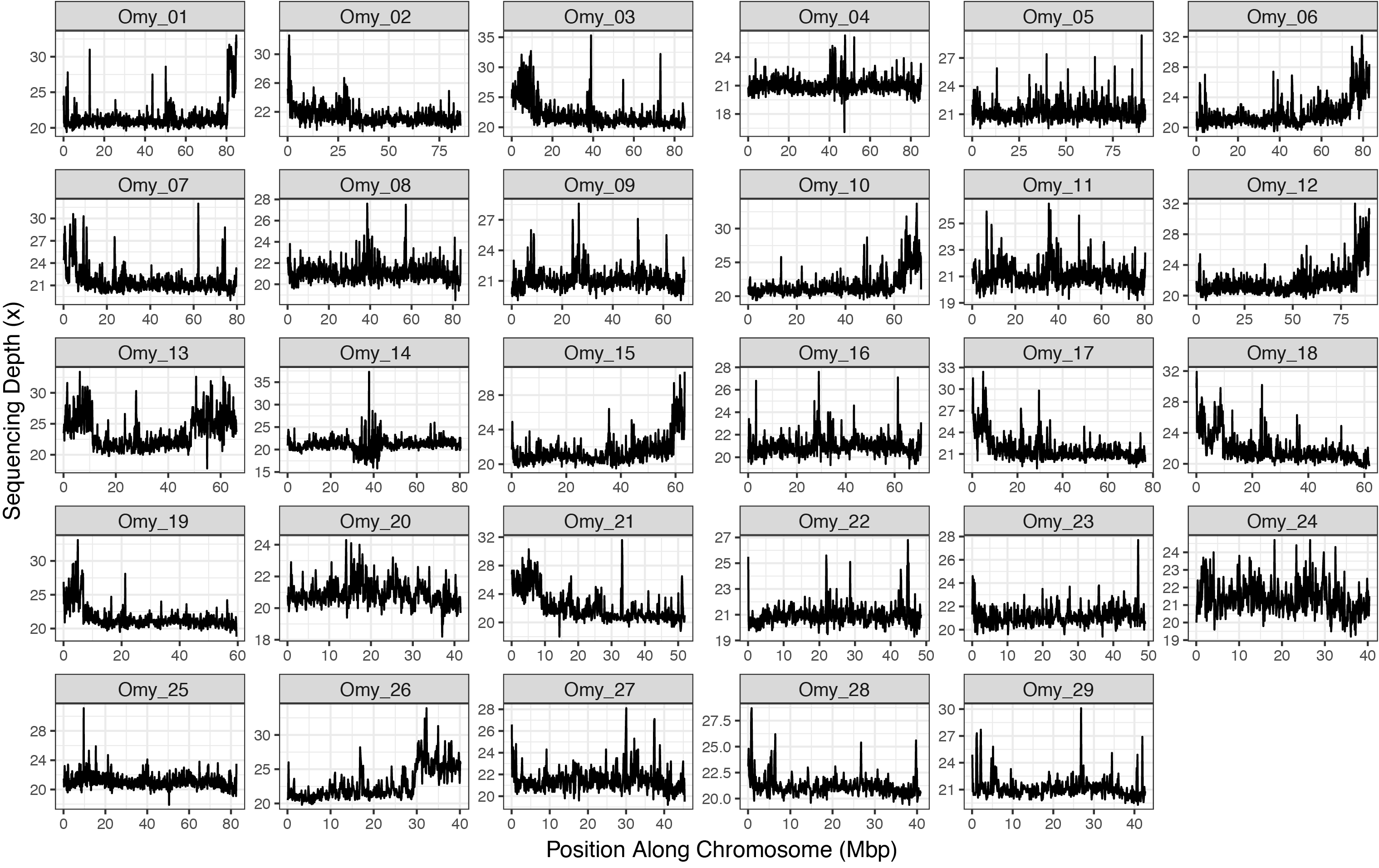


Figure S2. Sequencing depth (coverage) by chromosome and per 100,000bp window, averaged across piscivore and insectivore pools. Note the variable *x*- and *y*-axes.


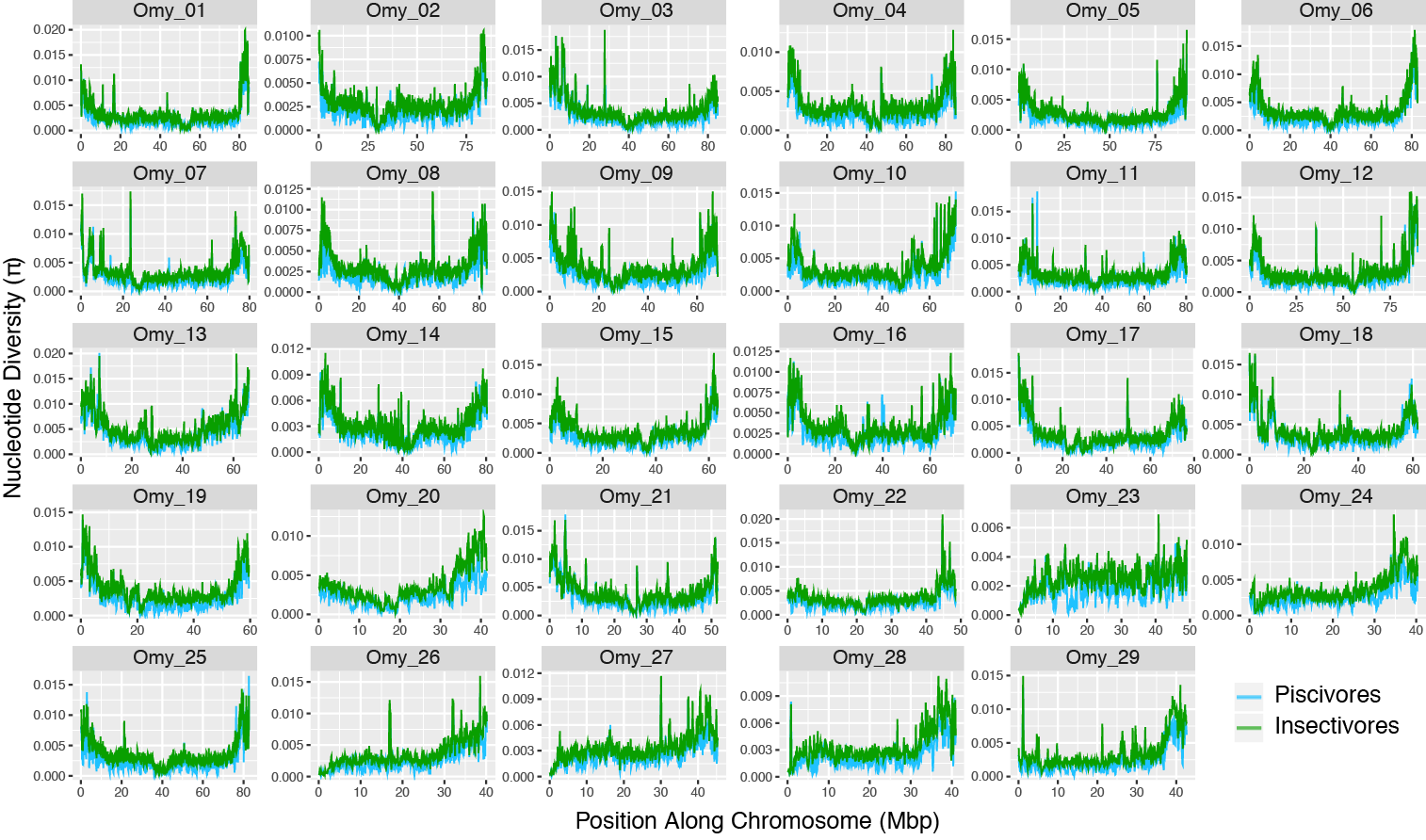


Figure S3. Nucleotide diversity by chromosome and per 100,000bp window of piscivorous and insectivorous rainbow trout from Kootenay Lake. Note the variable *x*- and *y*-axes.


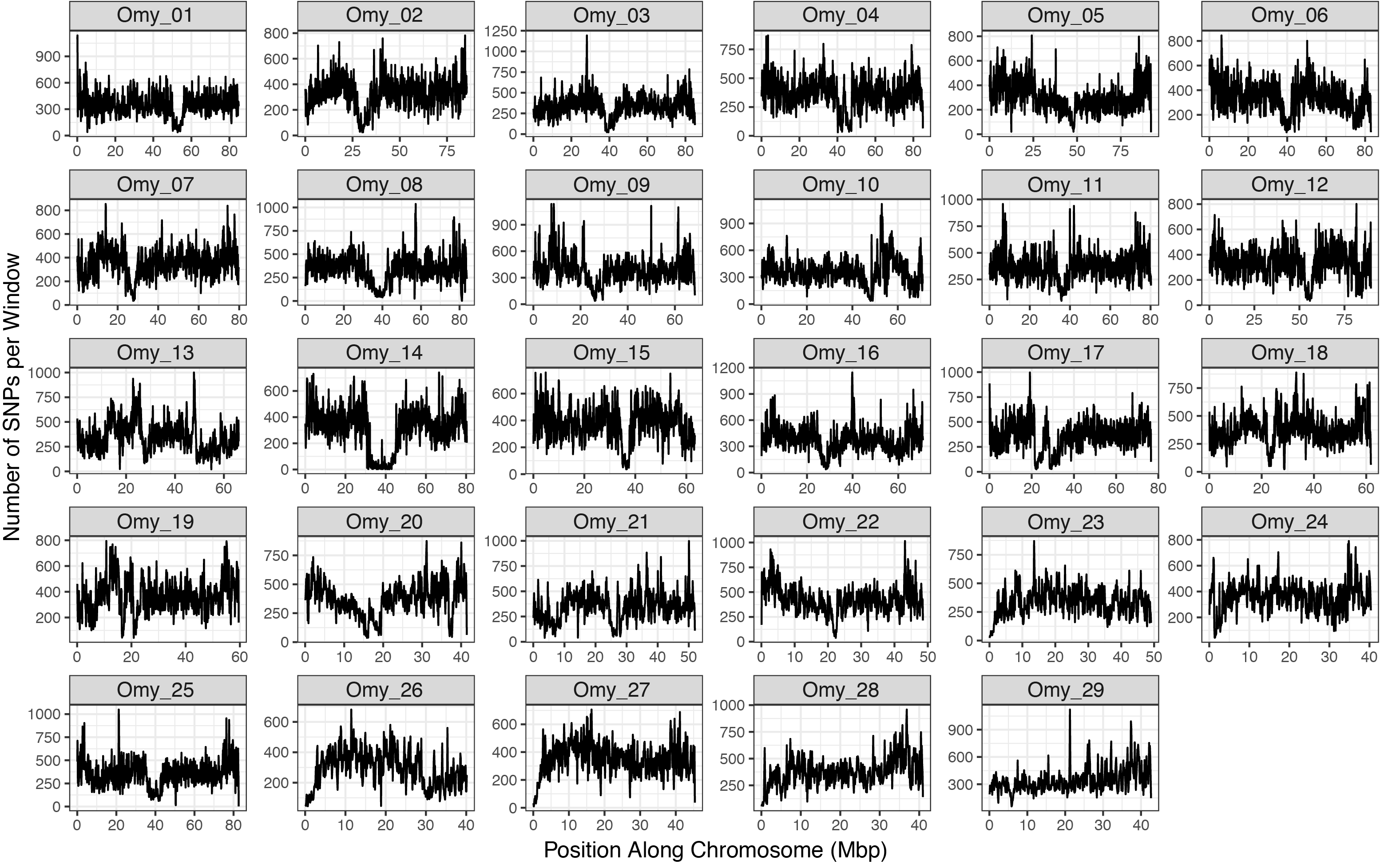


Figure S4. SNP density by chromosome and per 100,000bp window between piscivorous and insectivorous rainbow trout from Kootenay Lake. Note the variable *x*- and *y*-axes.

Figure S5. Distribution of single SNP Weir and Cockerham *F*_ST_ estimates after MAF and quality filtering for piscivores ("Gerrards") vs. insectivores ("non-Gerrards") in Kootenay Lake.

Figure S6. Distribution of -log_10_(FLK *p*-value) for SNPs from a single simulated chromosome, showing a non-uniform distribution.

Figure S7. Distribution of -log_10_(LK *p*-value) for SNPs from a single simulated chromosome, showing a non-uniform distribution.

Figure S8. Gene ontology (GO) enrichment analysis results for loci found in genomic windows with *F*_ST_ estimates greater than 0.2 (*n* = 7,219 windows) when the target gene set is not included in the background set. Processes are grouped by functional class (colour), and size of each process is scaled by the significance (log_10_ *p*-value) of enrichment of that process in relation to all processes in the genome.

Figure S9. Gene ontology (GO) enrichment analysis results for loci found in genomic windows with *F*_ST_ estimates greater than 0.3 (*n* = 2,737 windows) when the target gene set is not included in the background set. Processes are grouped by functional class (colour), and size of each process is scaled by the significance (log_10_ *p*-value) of enrichment of that process in relation to all processes in the genome.

Figure S10. Gene ontology (GO) enrichment analysis results for loci found in genomic windows with *F*_ST_ estimates greater than 0.4 (*n* = 854 windows) when the target gene set is not included in the background set. Processes are grouped by functional class (colour), and size of each process is scaled by the significance (log_10_ *p*-value) of enrichment of that process in relation to all processes in the genome.

Table S1. Locality information for individuals included in this study. Eighty individuals were sampled from each of the Blackwater and Gerrard sites, whereas the other sites were represented by 10 individuals each.

| **Site** | **Piscivore or Insectivore** | **Latitude** | **Longitude** |
| --- | --- | --- | --- |
| Blackwater River | I | 53.13425 | -123.59189 |
| Gerrard | P | 50.50947 | -117.27607 |
| Corn Creek | I | 49.09290 | -116.61144 |
| Crawford Creek | I | 49.68683 | -116.81820 |
| Cultus Creek | I | 49.32534 | -116.78876 |
| Goat River | I | 49.07408 | -116.50724 |
| Hendryx Creek | I | 49.76851 | -116.85717 |
| La France Creek | I | 49.52654 | -116.78181 |
| Tam O'Shanter Creek | I | 49.79215 | -116.84807 |
| Redfish Creek | I | 49.61315 | -117.04604 |
| Sanca Creek | I | 49.37488 | -116.72954 |
| Summit Creek | I | 49.14942 | -116.60710 |

Table S2. Genomic position and *F*_ST_ estimates of the twenty most divergent 100,000bp windows between piscivorous and insectivorous rainbow trout in Kootenay Lake.

| **Chromosome** | **Position** | **# SNPs** | | ***F*_ST_** | |
| --- | --- | --- | --- | --- | --- |
| 2 | 30950000 | 61 | 0.746 | |  |
| 1 | 20450000 | 221 | 0.658 | |  |
| 27 | 45350000 | 37 | 0.654 | |  |
| 17 | 56050000 | 331 | 0.647 | |  |
| 1 | 20550000 | 175 | 0.641 | |  |
| 7 | 2450000 | 273 | 0.635 | |  |
| 25 | 56950000 | 218 | 0.634 | |  |
| 16 | 60250000 | 125 | 0.633 | |  |
| 5 | 53250000 | 221 | 0.632 | |  |
| 23 | 16450000 | 356 | 0.628 | |  |
| 17 | 36050000 | 271 | 0.623 | |  |
| 3 | 64350000 | 212 | 0.622 | |  |
| 11 | 56650000 | 371 | 0.621 | |  |
| 21 | 33650000 | 605 | 0.620 | |  |
| 17 | 34950000 | 362 | 0.619 | |  |
| 16 | 35450000 | 306 | 0.617 | |  |
| 16 | 35550000 | 216 | 0.616 | |  |
| 5 | 55850000 | 181 | 0.611 | |  |
| 3 | 64250000 | 306 | 0.611 | |  |
| 19 | 36650000 | 437 | 0.609 | |  |

Table S3. Simulation results from local score analyses of FLK and LK test results.

| Statistic | FLK | LK |
| --- | --- | --- |
| Number of tests where exactly the same region was found | 551 | |
| Number of tests where the majority of region overlapped between tests, but not completely | 35 | |
| Number of tests when LK found a region that FLK didn't | 16 |  |
| Number of tests when FLK found a region that LK didn't |  | 30 |
| Percentage of time tests disagreed | 6.90% | |
| Minimum Number of significant outlier regions found in a genome | 1 | 0 |
| Maximum Number of significant outlier regions found in a genome | 13 | 13 |
| Maximum Number of chromosomes with significant outlier regions found in a genome | 11 | 11 |
| Average number of significant regions per genome | 5.92 | 5.79 |
| Average number of significant chromosomes per genome | 5.58 | 5.50 |
| **Type I error rate per genome (chromosome-level correction)** | **0.190** | **0.183** |

Table S4. Annotated genes in the five windows with highest *F*_ST_ estimates between piscivores and insectivores.

| Chromosome | Window | *F*_ST_ Rank | Locus | Locus Position | Protein |
| --- | --- | --- | --- | --- | --- |
| 2 | 30,900,000 - 31,000,000 | 1 | LOC110501635 | 30,387,211 - 30,917,034 | serine/threonine-protein kinase BRSK2-like |
| 2 | 30,900,000 - 31,000,000 | 1 | abtb2 | 30,924,465 - 31,075,877 | ankyrin repeat and BTB/POZ domain-containing protein 2 |
| 1 | 20,400,000 - 20,500,000 | 2 | LOC110510928 | 20,482,398 - 20,488,002 | DET1 homolog |
| 27 | 45,300,000 - 45,400,000 | 3 | N/A | N/A | N/A |
| 17 | 56,000,000 - 56,100,000 | 4 | LOC110494505 | 56,058,646 - 56,294,256 | protein FAM19A1-like isoform X1 |
| 1 | 20,500,000 - 20,600,000 | 5 | LOC110511054 | 20,501,960 - 20,505,784 | uncharacterized LOC110511054 |
| 1 | 20,500,000 - 20,600,000 | 5 | LOC110511247 | 20,518,985 - 20,537,093 | microtubule-associated proteins 1A/1B light chain 3B |
| 1 | 20,500,000 - 20,600,000 | 5 | LOC110511130 | 20,537,411 - 20,574,866 | zinc finger CCHC domain-containing protein 14-like |
| 1 | 20,500,000 - 20,600,000 | 5 | LOC110511358 | 20,587,209 - 20,619,848 | junctophilin-3-like isoform X1 |
